# Retrospective study of rare cutaneous malignant adnexal tumors of the head and neck in a tertiary care cancer hospital: a case series

**DOI:** 10.1186/s13256-017-1212-8

**Published:** 2017-03-12

**Authors:** Omer Waqas, Muhammad Faisal, Irfan Haider, Awais Amjad, Arif Jamshed, Raza Hussain

**Affiliations:** 1 Department of Pathology, Shaukat Khanum Memorial Cancer Hospital & Research Centre, Lahore, Pakistan; 2 Department of Surgical Oncology, Shaukat Khanum Memorial Cancer Hospital & Research Centre, 7-A, Block-R3, Johar Town, Lahore, Pakistan; 3 Department of Radiation Oncology, Shaukat Khanum Memorial Cancer Hospital & Research Centre, Lahore, Pakistan

**Keywords:** Adnexal carcinoma, Skin adnexal tumors, Head and neck cancers, Radiotherapy

## Abstract

**Background:**

Adnexal tumors of the skin are a large and diverse group of benign and malignant neoplasms, which exhibit morphological differentiation toward one of the different types of adnexal epithelium present in normal skin and they pose a diagnostic challenge. The purpose of this study is to share our experience with these rare but aggressive tumors at a tertiary care cancer hospital in a developing country. A retrospective review of 11 patients diagnosed with rare adnexal tumors and their variants from January 2005 to December 2014, treated either surgically or non-surgically, was performed to describe the clinicopathological characteristics and outcome of the disease.

**Case presentation:**

A total of 11 patients were diagnosed with adnexal carcinoma and its variants: a 34-year-old Sindhi man, a 59-year-old Punjabi woman, a 32-year-old woman from Khyber Pakhtunkhwa, a 43-year-old Punjabi woman, a 64-year-old Punjabi man, a 51-year-old man from Khyber Pakhtunkhwa, a 51-year-old Punjabi woman, a 74-year-old Punjabi woman, a 75-year-old Punjabi man, a 61-year-old man from Khyber Pakhtunkhwa, and a 53-year-old man from Khyber Pakhtunkhwa. The male to female ratio was 1.2:1. The histological variations were sebaceous differentiation (*n* = 4), microcystic adnexal carcinoma (*n* = 4), trichilemmal carcinoma (*n* = 1), pilomatrix carcinoma (*n* = 1), and hidradenocarcinoma (*n* = 1). The mean age at presentation was 54 years (range 32 to 75). The primary subsite of involvement was the scalp in nine patients followed by eyelids in two patients. Surgery was the primary treatment modality in almost all patients; postoperative radiotherapy (PORT) was offered to eight patients. The median dose of radiation was 45 Gy to the primary site. Indications for radiotherapy included close margins (*n* = 2), positive margins (*n* = 1), high grade histology (*n* = 4), and multifocal disease (*n* = 1). On follow-up, two patients presented with local, one regional and two patients developed distant metastasis.

**Conclusions:**

Adnexal carcinomas are rare tumors with diverse histological patterns and a tendency for locoregional and distant metastasis. Surgery should be the mainstay of treatment reserving radiotherapy for adjuvant, palliative, and re-treatment scenarios.

## Background

Cutaneous malignancies are generally categorized as melanoma and non-melanoma skin cancers. Non-melanoma tumors account for 93 to 96 % of skin cancers and melanoma tumors account for only 4 to 7 %. Both basal cell (75 %) and squamous cell carcinoma (20 %) make up the majority of non-melanoma skin tumors whereas benign and malignant adnexal tumors represent only 1 to 2 %. Adnexal carcinomas of the skin derive from structures that have a common origin such as the apocrine and eccrine sweat glands, sebaceous glands, and hair follicles [1]. Adnexal tumors represent a wide variety of neoplasms that vary in behavior and malignant potential and pose a diagnostic challenge for pathologists and surgeons. In terms of behavior, malignant skin adnexal tumors (SATs) are locally aggressive and have the potential for local and distant metastasis with poor outcome.

## Case presentation

All patients’ data were retrieved from the Cancer Registry Database of our hospital. Data were retrieved for patients who were histologically diagnosed as having SAT and its variants. Demographic data for each individual including age at diagnosis, sex, risk factors, grade, stage, geographic location were all obtained from the same database. All patients had a baseline computed tomography (CT) scan or magnetic resonance imaging (MRI) of the primary site. Findings of these imaging modalities were noted. The treatment modalities offered to the patients were recorded. Total duration of follow-up and status at last follow-up were also recorded. The study was exempted by the Institutional Review Board (IRB).

A total of 11 patients were diagnosed with adnexal tumors and its variants. The male to female ratio was 1.2:1. The histological variations were sebaceous differentiation (*n* = 4), microcystic adnexal carcinoma (*n* = 4), trichilemmal carcinoma (*n* = 1), pilomatrix carcinoma (*n* = 1), and hidradenocarcinoma (*n* = 1). The mean age at presentation was 54 years (range 32 to 75). The primary subsite of involvement was the scalp in nine patients followed by eyelids in two patients. Surgery was the primary treatment modality in almost all patients; postoperative radiotherapy (PORT) was offered to eight patients. The median dose of radiation was 45 Gy to the primary site. Indications for radiation therapy included close margins (*n* = 2), positive margins (*n* = 1), high grade histology (*n* = 4), and multifocal disease (*n* = 1). On follow-up, three patients developed locoregional recurrence (2 local and 1 regional) and two patients developed distant metastasis. None of the patients who received radiation therapy in a postoperative setting developed locoregional recurrence (Table 1).

**Table 1:** Patient characteristics and treatment outcome

Age (In Years)	Sex	Subsite	Surgery	Ethnicity	Local recurrence	Regional recurrence	Histopathology	RT dose	Distant metastasis
34	M	Scalp	Yes	Sindhi	No	No	Sebaceous carcinoma well differentiated	45 Gy/10 #	
59	F	Scalp	Yes	Punjabi	No	No	Microcystic adnexal carcinoma	45 Gy/10 #	
32	F	Scalp	Yes	Pakhtun	No	No	Pilomatrix carcinoma	45 Gy/10 #	
43	F	Scalp	Yes	Punjabi	Yes	Yes	Sebaceous carcinoma		
64	M	Scalp	Yes	Punjabi			Hidradenocarcinoma	45 Gy/10 #	Yes
51	M	Scalp	Yes	Pakhtun	No	No	Trichilemmal carcinoma		
51	F	Eyelid	Yes	Punjabi	No	Yes	Sebaceous carcinoma poorly differentiated	45 Gy/10 #	
74	F	Eyelid	Yes	Punjabi	No	No	Sebaceous carcinoma poorly differentiated		
75	M	Scalp	Yes	Punjabi			Adnexal carcinoma	60 Gy/30 #	Yes
61	M	Scalp	Yes	Pakhtun	Yes	No	Adnexal carcinoma	50 Gy/20 #	
53	M	Scalp	Yes	Pakhtun	No	No	Microcytic adnexal carcinoma	45 Gy/10 #	

# fractions, *F* female, *M* male, *RT* radiotherapy

### Case 1

A 34-year-old man from Sindh presented with a lesion on his scalp for 1 year which was excised at another hospital. Histopathology showed sebaceous carcinoma with close margin. He was offered radiotherapy 45 Gy in 10 fractions. He had no recurrence at his 2-year follow-up.

### Case 2

**Fig. 1:**
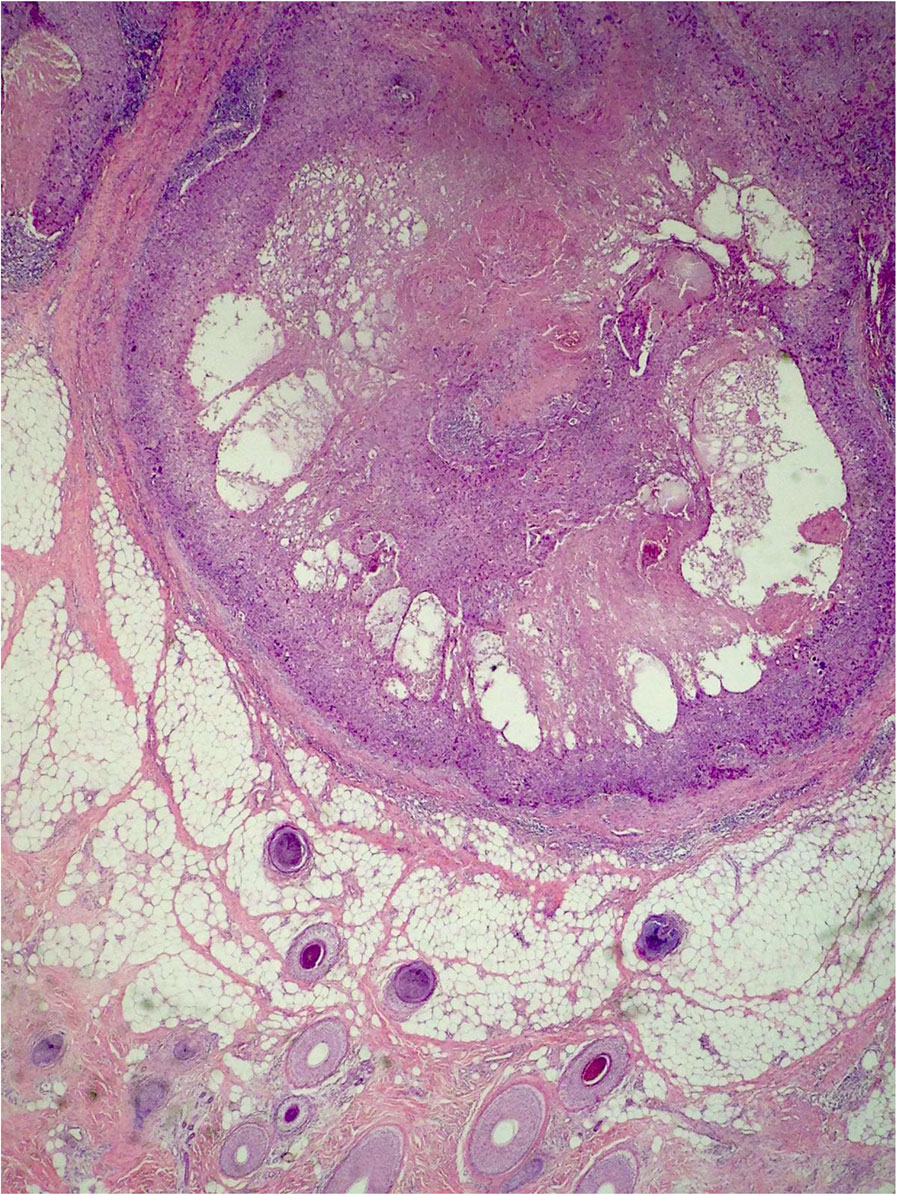
Microcystic adnexal carcinoma: Deep dermis showing infiltrating tubules and cords with extension into the panniculus

**Fig. 2:**
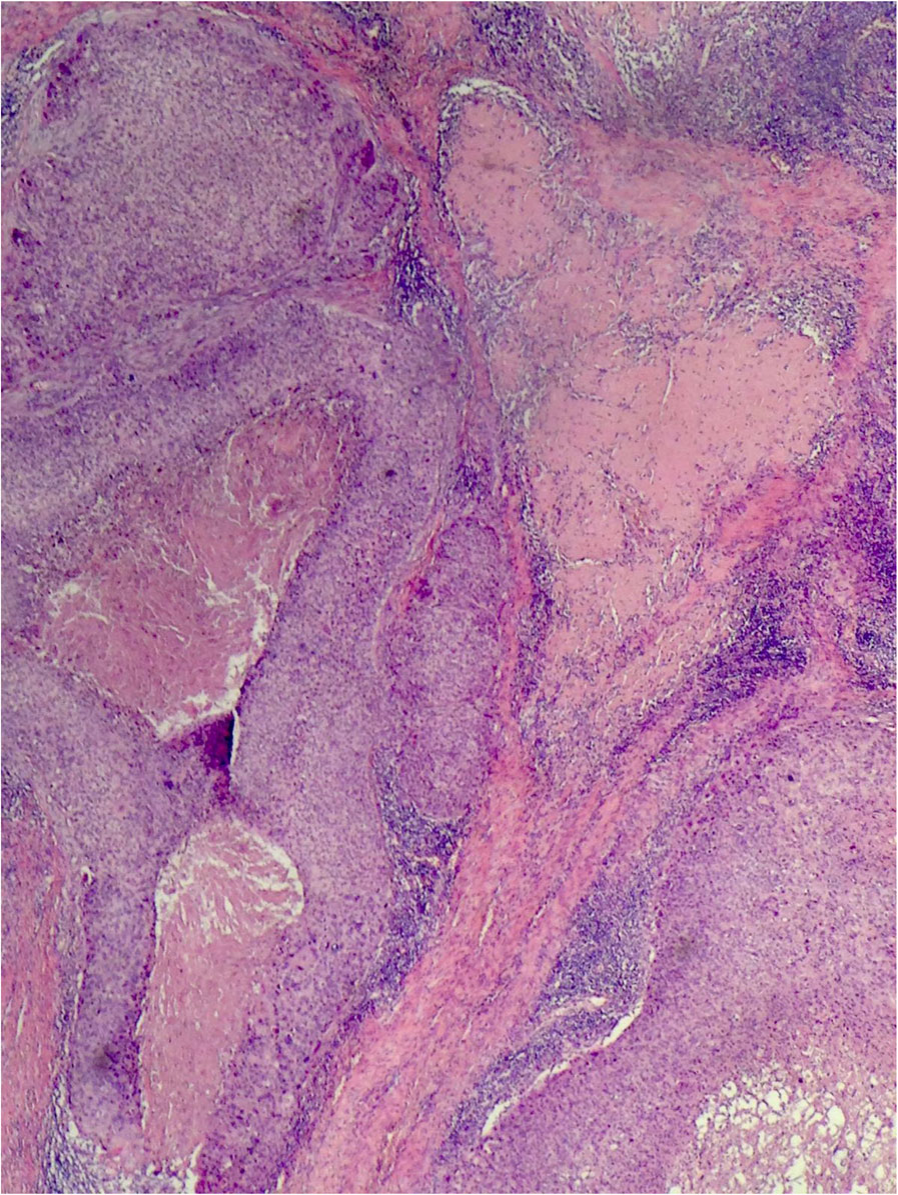
Microcystic adnexal carcinoma: Infiltrating tubules and cords in between the hair follicles

A 59-year-old woman from Punjab presented with a microcystic adnexal carcinoma on her forehead for more than 1 year, which had been operated on at an outside hospital and had a positive surgical margin. She was treated with radiotherapy 45 Gy in 10 fractions. She had no recurrence at her 3-year follow-up (Figs. 1 and 2).

### Case 3

**Fig. 3:**
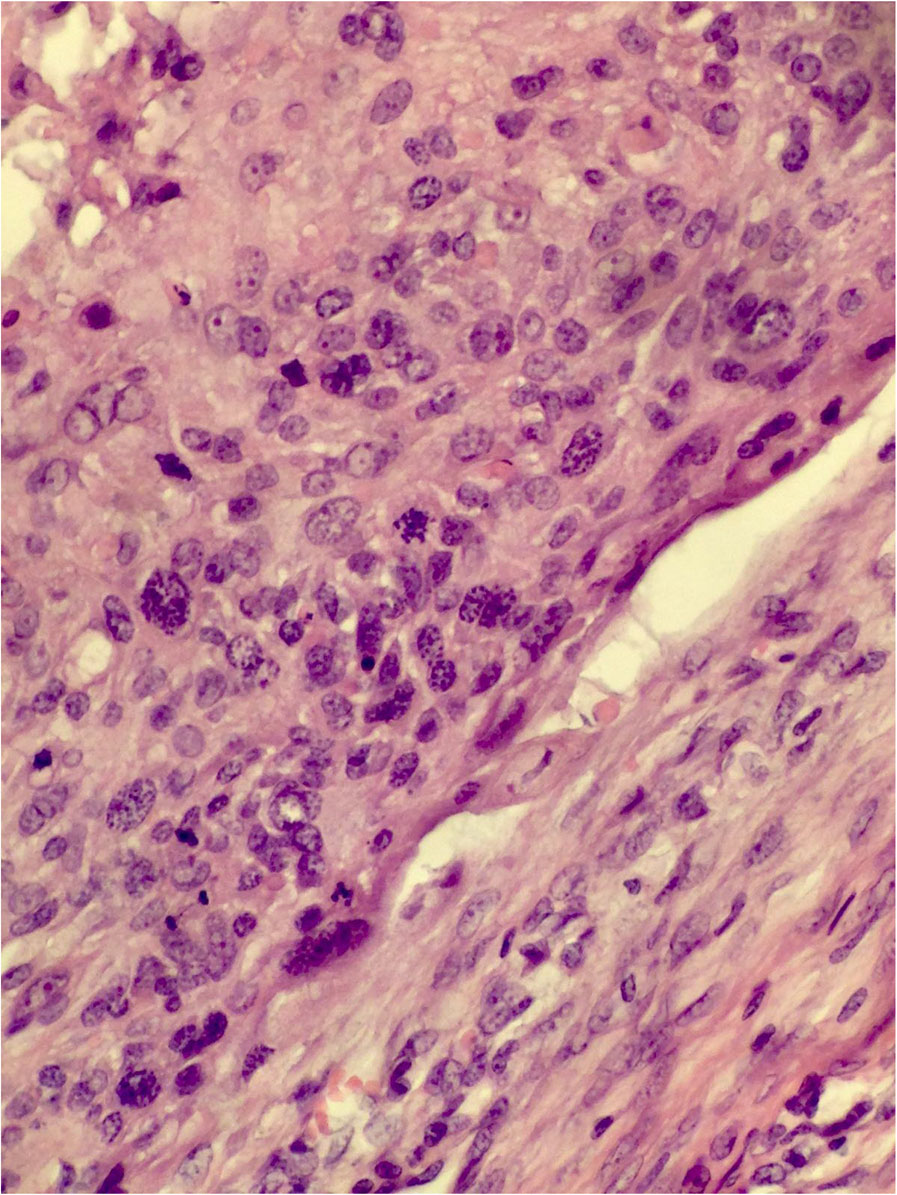
Pilomatrix carcinoma: Island and tumor with central cystic spaces located within the subcutaneous fat

**Fig. 4:**
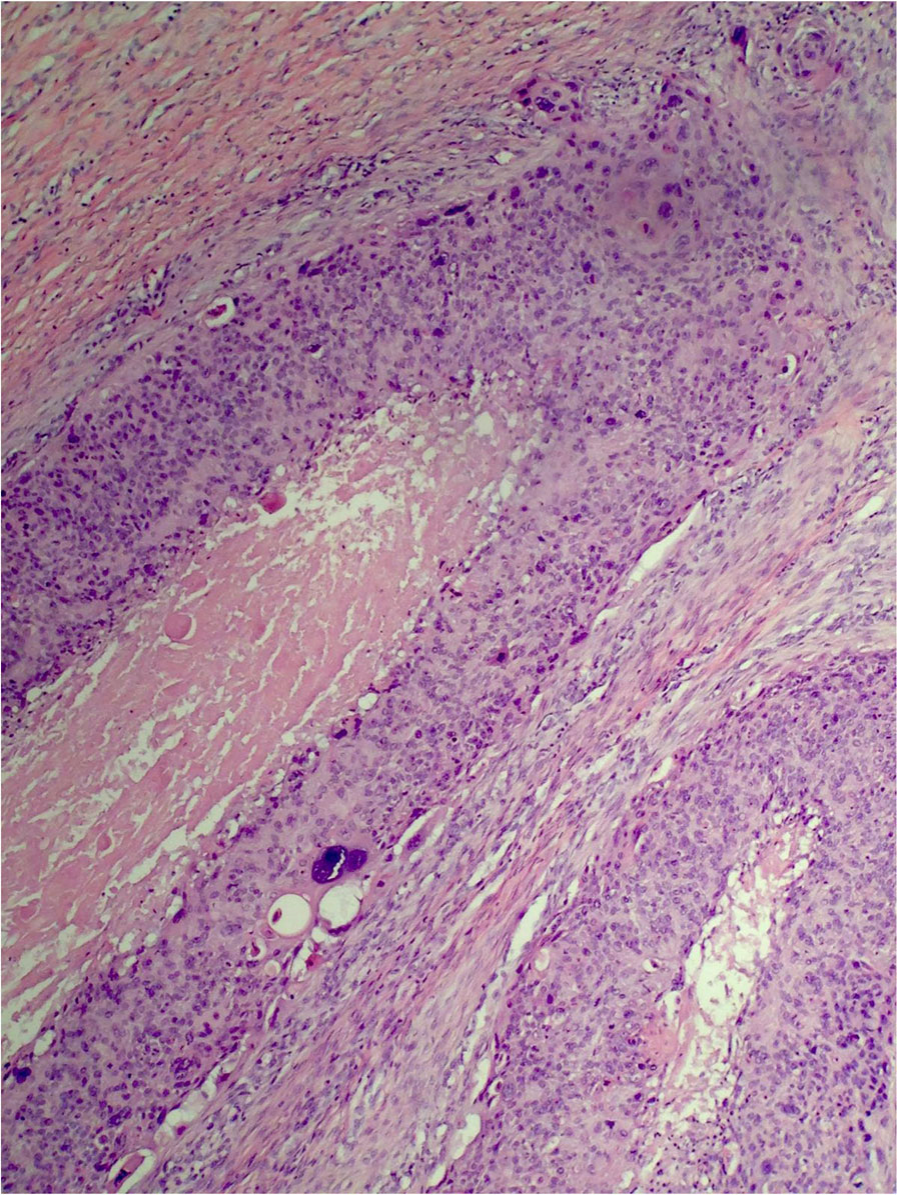
Pilomatrix carcinoma: Large tumor nests containing keratin debris in the center. Note the adjacent desmoplastic stroma

A 32-year-old woman from Khyber Pakhtunkhwa presented with a biopsy-proven pilomatrix adnexal carcinoma on the occipital region of her scalp which had been present for 2 to 3 years. A wide local excision was done along with split thickness skin graft reconstruction. Histopathology showed a positive margin, so radiotherapy was given at 45 Gy in 10 fractions. She was disease free at her 3-year follow-up without recurrence (Figs. 3 and 4).

### Case 4

**Fig. 5:**
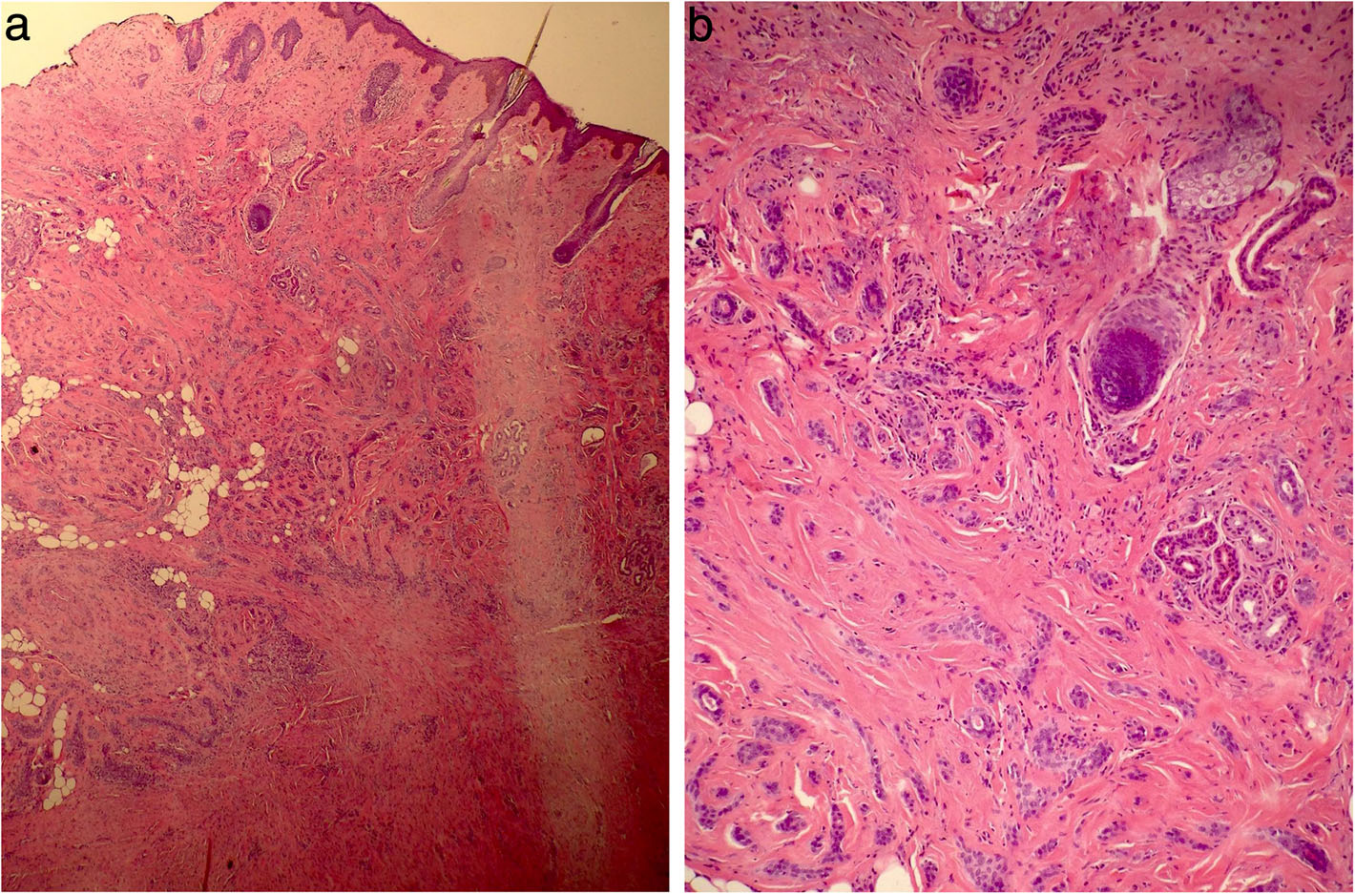
**a** Deep dermis with infiltrating cords and tubules. **b** Infiltrating tubules and cords in between hair follicles

A 43-year-old woman from Punjab presented with ulcerative growth at the vertex of her scalp with palpable occipital node, which she had had for more than 2 years. A biopsy showed high grade adnexal carcinoma (Fig. 5a, b). A wide local excision along with occipital lymph node clearance was done. Histopathology showed margins free of tumor but nodal involvement. She was offered radiotherapy but lost to follow-up. She returned to the clinic after 6 months with decreased vision and diplopia. A CT scan showed intracranial extension of the disease. She was given a Quad Shot regimen (14 Gy in 4 fractions) with palliative intent but showed no significant improvement. She is alive with disease and is on palliative care only.

### Case 5

A 64-year-old man from Punjab presented with a 4 to 5 cm mass on the left parietal region of his scalp, which he had for 6 to 7 years with occasional bleeding in another. A biopsy showed hidradenocarcinoma. A wide local excision was done with skin graft reconstruction. Histopathology showed clear margins. PORT at 45 Gy in 10 fractions was given. He presented again with pain in his right iliac fossa. A CT scan showed pulmonary and bone metastasis involving his tibia. Currently he is alive with disease and is under palliative care.

### Case 6

A 51-year-old man from Khyber Pakhtunkhwa presented with two scalp swellings which a biopsy revealed to be trichilemmal carcinoma. A wide local excision was done with skin graft reconstruction. No adjuvant treatment was offered. He was alive at his 4-year follow-up.

### Case 7

A 51-year-old woman from Punjab presented with biopsy-proven poorly differentiated sebaceous cell carcinoma on her eyelid for 2 years. The carcinoma was excised at another hospital. We offered PORT at 45 Gy in 10 fractions. She developed regional recurrence involving her right parotid and neck node at level 1. A right radical parotidectomy and neck dissection levels 1 to 4 was done. She was re-irradiated with 60 Gy in 30 fractions to her parotid and neck. She had no disease at her 5-year follow-up.

### Case 8

A 74-year-old woman from Punjab presented with a small nodule on her left eyelid for 2 months. A wide local excision was done. Histopathology showed adnexal carcinoma (a poorly differentiated sebaceous carcinoma) with clear margins but she was lost to follow-up for radiation therapy.

### Case 9

A 75-year-old man from Punjab presented with a biopsy-proven adnexal carcinoma with extensive destruction of skull bones and intracranial extension. Due to inoperability, he was offered radiotherapy at 60 Gy in 30 fractions. The disease remained stable following radiotherapy for 2 years which was assessed on repeat MRI scans. After 2 years, he developed spine metastasis. He is on palliative care only at the moment.

### Case 10

**Fig. 6:**
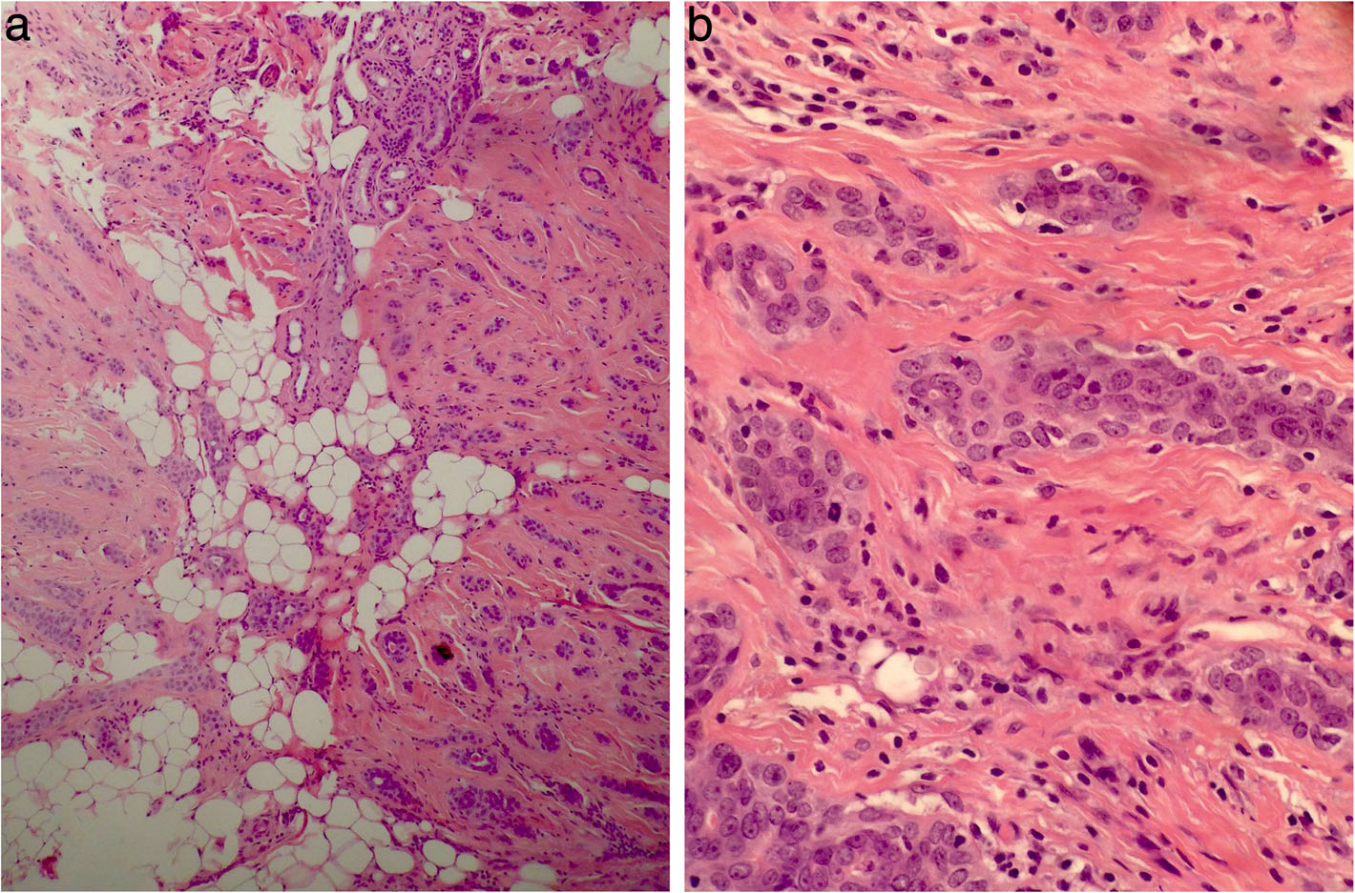
Infiltrating pattern of adnexal carcinoma. **a** Extension into panniculis. **b** Tumor composed of cords and tubules of round to oval cells

A 61-year-old man from Khyber Pakhtunkhwa presented with an adnexal tumor at left temporoparietal area which was operated on at another hospital with unknown margins. He was treated with radiotherapy at 50 Gy in 20 fractions but developed local recurrence involving orbital and intracranial extension. He is currently under palliative care only (Fig. 6a, b).

### Case 11

A 53-year-old man from Khyber Pakhtunkhwa presented with left temporal scalp lesion for 12 years. A wide local excision was done. Histopathology showed microcystic adnexal carcinoma with positive margin. He was treated with radiotherapy at 45 Gy in 10 fractions. He had no recurrence at his 3-year follow-up.

## Discussion

SATs are a large and diverse group of benign and malignant neoplasms, which exhibit morphological differentiation toward one of the different types of adnexal epithelium present in normal skin: pilosebaceous unit, eccrine, and apocrine. Adnexal tumors may display more than one line of differentiation (hybrid/composite tumors), rendering precise classification of these neoplasms difficult [2]. The diagnosis of these mixed adnexal tumors relies on histological evaluation; they are usually classified according to the predominant morphological component [3].

The etiology of malignant adnexal carcinomas is unknown; however, ultraviolet light and radiation have been implicated in their pathogenesis [4]. However, most of the patients in our series have no such exposure to radiation except two patients who were farmers by profession and had a history of sunlight exposure for long hours. The differential diagnosis includes desmoplastic trichoepithelioma, syringoma, trichoadenoma, morpheaform basal cell carcinoma, squamous cell carcinoma, and metastatic breast carcinoma [5]. Immunohistochemistry can help distinguish malignant adnexal carcinomas from other tumors, and highlights eccrine and pilar differentiation [6]. Two of our patients’ specimens stained positive for cytokeratin: one for p63 and one for CAM 5.2.

Rajalakshmi and Correa reported 21 cases of SATs with a male to female ratio of 1.1:1 [7]. Wang *et al*. reported a series of nine cases with a mean age of 67 [8]; the mean age of our patients was 54 years with none of the patients aged below 30 years. The histological diversity between the series of Wang *et al*. [8] and our series was quite similar with sebaceous differentiation, microcystic adnexal carcinoma, trichilemmal carcinoma, pilomatrix carcinoma, and hidradenocarcinoma being the most commonly presented variations.

Malignant adnexal carcinoma is a poorly circumscribed, deeply infiltrative, asymmetric tumor [9]. Surgery with wide local excision is the mainstay treatment modality for adnexal tumors [10]. Because of subtle clinical findings and banal appearance, it can grow undiagnosed for years. Comprehensive marginal excision is needed because of the widespread infiltration and indistinct boundaries of the disease. In our case series, almost all the cases were treated with surgery while PORT was only offered to those with close (<5 mm) margins, positive margins, and high grade histology. The role of radiation therapy has been undefined in SATs but Wang *et al*. show good locoregional control (100 %) with radiotherapy in a postoperative setting [8]. The median dose that we offered in our hospital was 45 Gy in 10 fractions in a hypofractionated course (more than 2 Gy/day) as compared to 55 Gy given by Baxi *et al*. in a series of 14 cases with 100 % locoregional control. Stein *et al*. suggested that definitive radiotherapy for adnexal carcinomas has resulted in a clinically aggressive transformation which we cannot comment on in our study as none of the patients received radical radiotherapy; however, one patient who did not have radiotherapy post-tumor resection developed locoregional recurrence [11, 12].

Currently no recommended guidelines are available regarding the role of adjuvant radiotherapy in malignant adnexal carcinomas, yet the role of adjuvant radiotherapy has been shown in a number of case series in the background of adverse features, such as close or positive margin, high grade of the tumor, and perineural invasion.

This case series has highlighted multiple aspects of malignant adnexal carcinomas, such as clinical aspects, treatment modalities used, locoregional failure, and histopathological diversity, but it has a few limitations such as short follow-up, a study sample that is small for comment on the epidemiology of the disease, and no patients were treated with definitive radiotherapy to evaluate its role as a single modality.

## Conclusions

Adnexal tumors are rare tumors with diverse histological patterns with a tendency for locoregional and distant metastasis. Surgery should be the mainstay of treatment reserving radiotherapy for adjuvant, palliative, and re-treatment scenarios. Studies from multiple centers will help to establish guidelines to address these rare but aggressive tumors.

## Abbreviations

CT: Computed tomography
MRI: Magnetic resonance imaging
PORT: Postoperative radiotherapy
SAT: Skin adnexal tumor

## References

[1] Güerrissi JO, Quiroga JP. Adnexal carcinomas of the head and neck. Indian J Plast Surg. 2008;41(2):229–34. 10.4103/0970-0358.44950 PMC2740523 19753272

[2] Rudolph P. Benign adnexal skin tumors. Pathologe. 2002;23:71–8. 10.1007/s00292-001-0506-0 11974506

[3] Rodriguez‐Diaz E, Armio M. Mixed tumors with follicular differentiation: complex neoplasms of the primary epithelial germ. Int J Dermatol. 1995;34:782–5. 10.1111/j.1365-4362.1995.tb04397.x 8543411

[4] Abbate M, Zeitouni NC, Seyler M, *et al*. Clinical course, risk factors, and treatment of microcystic adnexal carcinoma: a short series report. Dermatol Surg. 2003;29:1035–8. 10.1046/j.1524-4725.2003.29296.x 12974700

[5] Leibovitch I, Huilgol SC, Selva D, *et al*. Microcystic adnexal carcinoma: treatment with Mohs micrographic surgery. J Am Acad Dermatol. 2005;52:295–300. 10.1016/j.jaad.2004.10.868 15692477

[6] Friedman PM, Friedman RH, Jiang SB, *et al*. Microcystic adnexal carcinoma: collaborative series review and update. J Am Acad Dermatol. 1999;41:225–31. 10.1016/s0190-9622(99)70053-8 10426893

[7] Rajalakshmi T, Correa M. Clinicopathologic analysis of 21 cases of nevus sebaceous: a retrospective study. Indian J Dermatol Venereol Leprol. 2008;74:625–7. 10.4103/0378-6323.45107 19171988

[8] Wang LS, Handorf EA, Wu H, Liu JC, Perlis CS, Galloway TJ. Surgery and adjuvant radiation for high-risk skin adnexal carcinoma of the head and neck. Am J Clin Oncol. 2015. doi:10.1097/COC.0000000000000178. PMC4504824 25599317

[9] Goldstein DJ, Barr RJ, Santa Cruz DJ. Microcystic adnexal carcinoma: a distinct clinicopathological entity. Cancer. 1982;50:566–72. 10.1002/1097-0142(19820801)50:3<566::aid-cncr2820500330>3.0.co;2-q 7093897

[10] Yu JB, Blitzblau RC, Patel SC, *et al*. Surveillance, epidemiology and end results (SEER) database analysis of microscopic adnexal carcinoma (sclerosing sweat duct carcinoma) of the skin. Am J Clin Oncol. 2010;33:125–7. 10.1097/COC.0b013e31819791eb 19675445

[11] Baxi S, Deb S, Weedon D, Baumann K, Poulsen M. Microcystic adnexal carcinoma of the skin: the role of adjuvant radiotherapy. J Med Imaging Radiat Oncol. 2010;54(5):477–82. 10.1111/j.1754-9485.2010.02200.x 20958947

[12] Stein JM, Ormsby A, Esclamado R, Bailin P. The effect of radiation therapy on microcystic adnexal carcinoma: a case report. Head Neck. 2003;25(3):251–4. 10.1002/hed.10167 12599293

